# Meows encode less individual information than purrs and show greater variability in domestic than in wild cats

**DOI:** 10.1038/s41598-025-31536-7

**Published:** 2025-12-09

**Authors:** Danilo Russo, Anja Birgit Schild, Mirjam Knörnschild

**Affiliations:** 1 Laboratory of Animal Ecology and Evolution (AnEcoEvo), Dipartimento di Agraria, Università degli Studi di Napoli Federico II, Portici (Napoli), Italy; 2 Museum für Naturkunde, Leibniz-Institute for Evolution and Biodiversity Science, Berlin, Germany; 3 Animal Behaviour, Institute for Biology, Freie Universität Berlin, Berlin, Germany; 4 Evolutionary Ethology, Institute for Biology, Humboldt-Universität zu Berlin, Berlin, Germany

**Keywords:** Felidae, Individual signature, Domestication, Vocal communication, Ecology, Ecology, Evolution, Zoology

## Abstract

Domestication has shaped animal vocal behaviour, increasing flexibility and responsiveness to humans. In domestic cats (*Felis catus*), two vocalisations, meows and purrs, have distinct communicative roles. Meows are context-dependent signals primarily directed at humans; purrs are stereotyped, low-frequency sounds produced in affiliative contexts. Vocal individuality, key in mammalian communication, supports social recognition and interaction, but its presence across cats’ call types remains poorly understood. We examined whether cats encode individual identity in meows and purrs, hypothesising that meows might show stronger signatures due to their human-directed nature. We analysed 276 meows from 14 cats and 557 purrs from 21 cats. Both call types carried sufficient individual information, but purrs had significantly higher classification accuracy (84.6%) and encoded more information content (4.47 bit) than meows (63.2%, 2.65 bit). To place individuality in a domestication framework, we compared domestic cat meows with those of five wild relatives: African wildcat, European wildcat, jungle cat, cheetah, and cougar. Domestic cat meows showed greater acoustic dispersion than those of wild cats, reflecting increased vocal plasticity through domestication. These findings demonstrate how domestication has shaped feline vocalisations, with purrs acting as stable identity cues and meows emphasising flexibility over recognisability.

**Supplementary Information:**

The online version contains supplementary material available at 10.1038/s41598-025-31536-7.

## Introduction

 The signals animals use to communicate often encode differences linked to factors such as sex, age, body condition, or social group, as well as distinctive, stereotyped “individual signatures” specific to the emitter rather than to groups or categories^1^. Mostly, though not exclusively, vocal, individual signatures allow discrimination among unique individuals and play a critical role in the evolution of social processes, including kin recognition, cooperation, social learning, and the formation of social structures^1,2^. Therefore, individual recognition represents the most sophisticated level of recognition in animals^3^. Given the potential social benefits of recognising individuals, strong selective pressures likely drive the evolution and maintenance of individual distinctiveness in signals^4^.

Individuality in vocal signals has been extensively studied, and there is mounting evidence that many taxa carry individual signatures that are functionally relevant for communication^1,3,5^. Much less attention, however, has been paid to whether different call types within the same species vary in their ability to encode individual identity. The limited available evidence suggests that this variation exists and may have functional correlates. For example, in zebra finches (*Taeniopygia guttata*), contact calls encode stronger individual signatures than aggressive calls^6^. Distress and alarm calls convey weaker individual signatures than other call types in skuas^7^, putty-nosed monkeys^8^, and dwarf mongooses^9^. In bonobos, the strength of individual signatures decreases from calls produced in low-arousal contexts to those emitted in high-arousal situations^10^. Such comparisons are essential for understanding how identity information is distributed across a species’ vocal repertoire and how different call types vary in their ability to encode stable versus context-dependent information.

Since Darwin’s (1859) formulation of evolution by natural selection^11^, domestication and artificial selection have offered a compelling perspective on evolutionary processes. Comparing domesticated forms with their wild ancestors, as well as breeds or individuals within domestic species, can reveal consistent patterns of trait divergence and constraint, providing broad insights into evolution and its dynamics^12^. Like morphology and physiology, behaviour (including vocal behaviour) is also strongly influenced by domestication^13,14^. An additional layer of complexity in understanding how behaviour evolves in domestic animals is that it develops under the combined influence of domestication and interactions with humans, where signals that bridge the communicative gap between species are selected^15–18^.

Domestic cats (*Felis catus*) provide an excellent model to investigate how domestication and human interaction shape vocal behaviour and how individual identity is encoded across call types differing in structure, context, and evolutionary history. Once solitary animals that relied on long-range signals to communicate, domestic cats have adapted to life in close social groups, where direct interaction plays a crucial role^19,20^. Like dogs^21^, domestic cats have undergone profound changes in vocal behaviour through their long coexistence with humans^22^. Domestic cats exhibit a rich repertoire that includes call types directed at both conspecifics and humans^23^ and use their vocal flexibility to adjust communication strategies and elicit responses from caregivers^24^. The domestic cat’s vocal repertoire is extensive, with up to 21 distinct vocalisation types described in early work^23,25^ and further evidence from recent acoustic analyses supporting a complex, graded vocal system^26^. Differences between feral and domestic house cats suggest that socialisation with humans affects both the types of vocalisations produced and their acoustic characteristics^27^.

The purr and meow are two distinct vocalisations in the domestic cat’s repertoire, each serving different purposes and exhibiting unique characteristics. Purring is strictly defined as a continuous low-frequency, low-amplitude sound produced with the mouth closed on alternating egressive and ingressive airstreams (while breathing out and in), accompanied by body vibrations^28^. It occurs in ‘purring cats’ (all felids except the roaring species, with uncertainty for the snow leopard) and in the genet^ (family Viverridae)29^. While purr-like sounds have been described in other mammals, they do not meet this strict definition^28^. The fundamental frequency (F0) of purring, which ranges from 25 to 30 Hz, is much lower than that of most vocalisations^23,29,30^. Purring can last from less than a second to several minutes and may be combined with other tonal vocalisations^31,32^. Although cat purring has long been attributed to active muscle contractions (AMC), it may also be generated via standard myoelastic–aerodynamic (MEAD) mechanisms without neural input, with AMC likely acting as a modulatory rather than primary driver^33^.

Purring is often regarded as a low-arousal, affiliative signal in cats, linked with social contact and bonding, particularly between kittens and their mothers, for instance, broadcast by the former during suckling^23,34^. Yet purring is not limited to these contexts, as it also occurs in both solicitation and non-solicitation settings and even during stress, pain, or near death^23^. In cat–human communication, purring is used to maintain contact and promote calmness, as in cat–cat interactions^20^. Purrs may also be produced while soliciting food, in which case they embed a high-frequency element that humans perceive as particularly urgent^32^.

The meow is a highly versatile vocalisation whose acoustic structure varies with context, typically produced with an open mouth that gradually closes^23^. Its pitch, duration, and melody vary across contexts, with an F0 ranging from 208 to 1000 Hz^35^. Like most other cat vocalisations, meows are produced through the conventional MEAD mechanism, where self-sustained oscillations of the vocal folds arise from aerodynamic forces acting on the laryngeal tissues^33^. Meows are relatively rare in interactions among cats and may occur in contexts such as territorial disputes or mate attraction, and are more frequent in subadult life^23^. However, meows are common in cat–human communication, especially during play or when soliciting attention or food^19,27^.

Encoding individual signatures in domestic cat vocalisations has received limited attention. For instance, kittens’ isolation calls are individually distinct and vary between low- and high-arousal contexts^36^. Purr vocal parameters such as F0 and duration vary between individuals^28^, and meows convey information about the caller’s sex and identity^37^.

So far, no study has compared the strength of individual signatures between different cat vocalisations. Here, we examined meows and purrs to establish how individual identity is encoded in two vocalisations that differ in their acoustic structure, communicative context, and underlying production mechanisms. First, because meows are mostly used in human-directed communication, we hypothesise that they have evolved to convey clearer information about individual identity than purrs. We therefore predict that meows exhibit stronger individual vocal signatures than purrs. Second, we hypothesise that the selective pressures imposed by human–cat interactions during domestication favoured greater vocal flexibility in meows. We therefore predict that meows of domestic cats display higher within-species variability than those of their wild congeners.

## Materials and methods

### Data collection

We recorded cat vocalisations in 2020 and 2021 in twelve private households and two animal shelters in Berlin. Depending on the availability of the cats, each cat was recorded for 1 h each on one to five different days (on average, three days per cat). Recordings were made indoors in the context of food anticipation or other human attention (meows) and during petting sessions (purrs). We recorded vocalisations from 27 individual cats (**Table ****S1**), 10 females and 17 males, belonging to five different breeds (Birman, British Shorthair, European Shorthair, Neva Masquerade, Norwegian Forest Cat) and three different mixed breeds (European Shorthair Mix, Maine Coon Mix, Siam Mix). Except for one household with four cats, one or two animals lived in each participating household. A total of nine cats were housed in shelters, where they were kept either individually or in groups of up to three. All vocalizations used for analysis were collected in comparable human–cat interaction contexts, regardless of whether cats lived in private homes or shelters and regardless of group size.

**Fig. 1:**
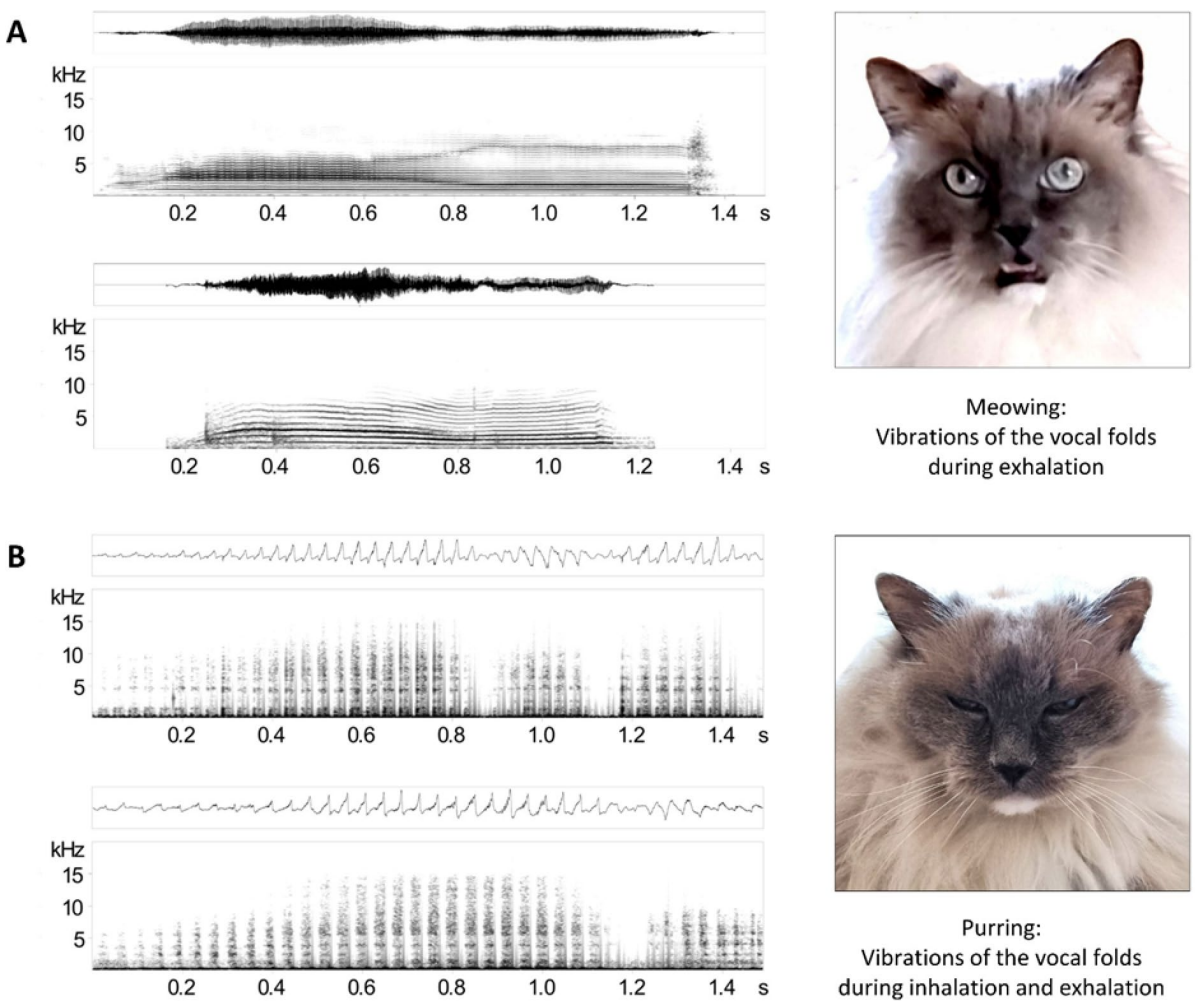
Spectrograms and oscillograms of (**A**) two meows and (**B**) two purrs. Oscillograms depict sound pressure over time, and spectrograms depict frequency over time. All spectrograms were created with a 1024-point FFT, 16-bit depth, and a Hamming Window with 87.5% overlap (sampling rate: 96 kHz, frequency resolution: 94 Hz, time resolution: 1.33 ms). The two pictures on the right depict Koda, a 15-year-old male Ragdoll cat, meowing and purring (credit: Marisa Idolo).

The vocalisations were recorded in stereo using a shotgun microphone (Sennheiser MKH 416-P48U3) and a WAV recorder (EDIROL by Roland, resolution: 16-bit and 96 kHz). The microphone was held as close as possible to the cat’s mouth, at 2–20 cm, with slight variation during recordings due to the animals’ movements. We converted stereo recordings into mono recordings using Avisoft SASLab Pro software (v5.2.14, R. Specht, Glienicke, Germany). We manually scanned recordings and stored meows and purrs separately for subsequent analyses (Fig. 1).

**Fig. 2:**
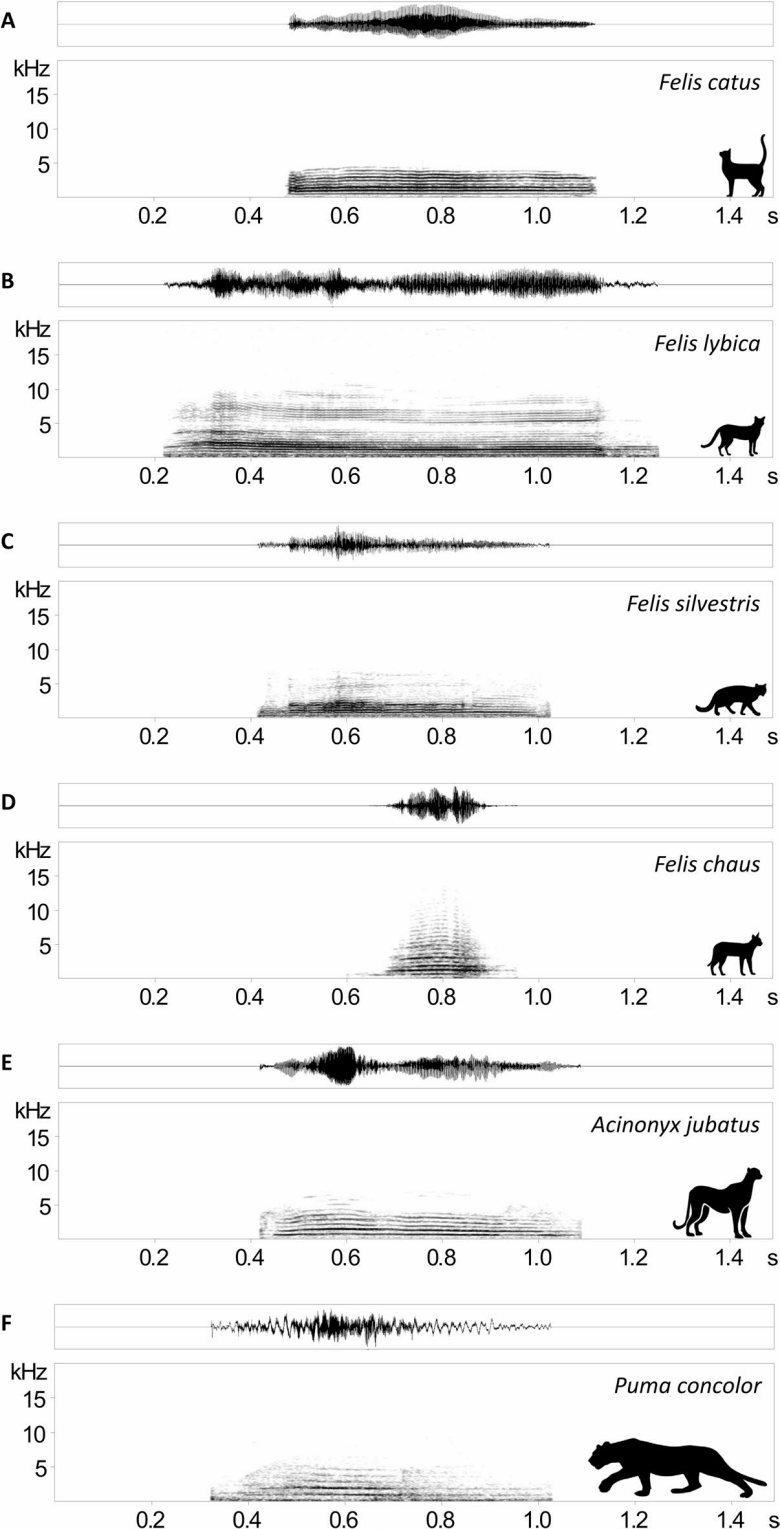
Spectrograms and oscillograms of meows from (**A**) the domestic cat, *Felis catus*, (**B**) the African wildcat, *Felis lybica*, (**C**) the European wildcat, *Felis silvestris*, (**D**) the jungle cat, *Felis chaus*, (**E**) the cheetah, *Acinonyx jubatus*, and (**F**) the cougar, *Puma concolor*.

We also extracted 185 meows of five additional cat species from the Animal Sound Archive of the Museum für Natural History in Berlin (Fig. 2): the jungle cat, *Felis chaus* (69 meows from five individuals), the African wildcat, *Felis lybica* (six meows from three individuals), the European wildcat, *Felis silvestris* (75 meows from six individuals), the cougar, *Puma concolor* (13 meows from three individuals), and the cheetah, *Acinonyx jubatus* (22 meows from three individuals). These recordings were used to contrast them with the meows of domestic cats.

### Acoustic analyses

We measured the duration and the spectral centroid of meows (for all 6 cat species) and purrs (for domestic cats) for a gross general characterisation of both vocalisation types. For statistical analyses, we extracted mel-frequency cepstral coefficients (MFCCs) from meows and purrs using a custom-built MATLAB routine (MATLAB’s `voicebox’ speech processing toolbox v. R2024b): the signal was divided into overlapping frames, a Fourier transform was computed for each frame, and the magnitude spectra were then passed through a mel-scaled filterbank of overlapping triangular filters. Finally, we took the logarithm of the filterbank energies and applied a discrete cosine transform to obtain the MFCCs.

MFCCs are spectral-based representations of entire signals and are widely used for human voice analysis and human speaker recognition (reviewed in^38,39^). They have also been used to analyse animal vocalisations^40,41^. MFCCs parameterize the spectral envelope of a signal, making them particularly well suited to capturing individual differences in broadband vocalizations. They are based on the mel-scale, which is linear up to 1 kHz and logarithmic above, resulting in a stronger emphasis on low frequencies^38,39^. We used the mel scale because cat vocalisations fall within the frequency range of human speech and hearing – the perceptual domain for which the mel scale was originally developed^23^. Due to the different lengths of meows and purrs, we used a sliding window frame of 30 ms for meows and 300 ms for purrs when extracting MFCCs. We subsequently averaged each MFCC value over all its frames for each meow and purr. In total, we extracted 10 MFCCs for meows and 10 MFCCs for purrs. All MFCC, as well as duration and spectral centroid for meows and purrs, are provided in the supporting information (**Data S1**).

### Statistical analyses

To test whether cats could be statistically discriminated based on their meows and purrs, we performed discriminant function analyses on four different data sets: (1) 276 meows from 14 cats (7–53 meows per cat); (2) 557 purrs from 21 cats (7–56 purrs per cat); (3) A balanced, reduced data set of 117 meows and 117 purrs from the same eight individuals (7–23 meows and purrs each per cat); and (4) 248 meows from six different cat species (N: 63 – *F. catus*; 69 – *F. chaus*, 6 – *F. lybica*, 75 – *F. silvestris*, 13 – *P. concolor*, 22 – *A. jubatus*). The data set (4) contained meows from 24 individuals: four *F. catus*, five *F. chaus*, three *F. lybica*, six *F. silvestris*, three *P. concolor*, and three *A. jubatus*.

DFAs were conducted separately for meows and purrs. For each DFA, we checked all parameters for multicollinearity and included them in a stepwise manner. We applied a cross-validation procedure (n-1) to estimate the correct classification success. We also adjusted the DFAs for the unequal number of cases per group by computing group sizes based on prior probabilities. We used binomial tests to assess whether the DFAs’ classification success was greater than expected by chance.

To assess the relative strength of individual signatures in meows and purrs, we calculated the information content encoded in each vocalisation type. We measured information content using the stereotypy index H_S_ (bits per signal), which represents the number of binary decisions necessary to discriminate among *N* individuals, following the quantitative method developed by Beecher (1989). We calculated type II MANOVAs (separately for meows and purrs) with 10 acoustic parameters (MCFF1-10) as dependent variables and individual identity as a random factor; we subsequently used the F statistic for each dependent variable in the following formula to calculate the stereotypy index H_S_:

H_s_ = log_2_ √((F + n-1)/n).

where *n* is the mean number of meows or purrs measured per individual (*n* = 14.7). We summed up contributions from each MFCC to estimate the total amount of encoded information in meows and purrs. Two to the power of *x* (2^*x*^) represents the number of unique combinations when *x* bits of information are available, so higher values of H_S_ indicate a greater potential for encoding information than lower values^42^.

Statistical tests were conducted in SPSS v29.0 (IBM Corporation, New York, USA) and RStudio 2022.12.0^43^. Significance was set at *P* < 0.05.

### Ethics

The sound recordings of *F. chaus*,* F. lybica*,* F. silvestris*,* P. concolor*, and *A. jubatus* came from the Animal Sound Archive of the Museum für Natural History in Berlin. The sound recordings from *F. catus* were made by A. Schild. Because data collection involved only non-invasive, passive sound recording of pet cats in their normal surroundings (private households, animal shelters) no animal experimentation permits were necessary under German law or institutional policy. Work was carried out in compliance with national legislation and university ethical standards, with written consent from cat and shelter owners. All methods are reported in accordance with the ARRIVE guidelines.

## Results

### Individual signatures in meows and purrs

Meows (*N* = 276; **Data S1**) had a mean duration of 0.72 s (range: 0.17–2.21 s) and a spectral centroid of 1738 Hz (range: 530–3680 Hz). Purrs (*N* = 557; **Data S1**), on the other hand, had a mean duration of 10.77 s (range: 1.09–39.82 s) and a spectral centroid of 268 Hz (range: 40–1550 Hz). Both meows and purrs encoded sufficient inter-individual variation to facilitate the statistical discrimination of individual cats. 65.2% of meows and 76.1% of purrs were classified to the correct individual (meows: 14 cats; purrs: 21 cats). The classification success obtained by both DFAs (see Table S2 for details on model fit and Table S3-S4 for the classification matrices) was significantly higher than expected from a random classification (binomial tests: *p* < 0.001; 7.14% chance level for meows; 4.76% chance level for purrs). This suggests that purrs encode a stronger individual signature than meows, but the data sets contained different numbers of individuals, making direct comparisons challenging.

### Comparing the signature strength in meows and purrs

**Fig. 3:**
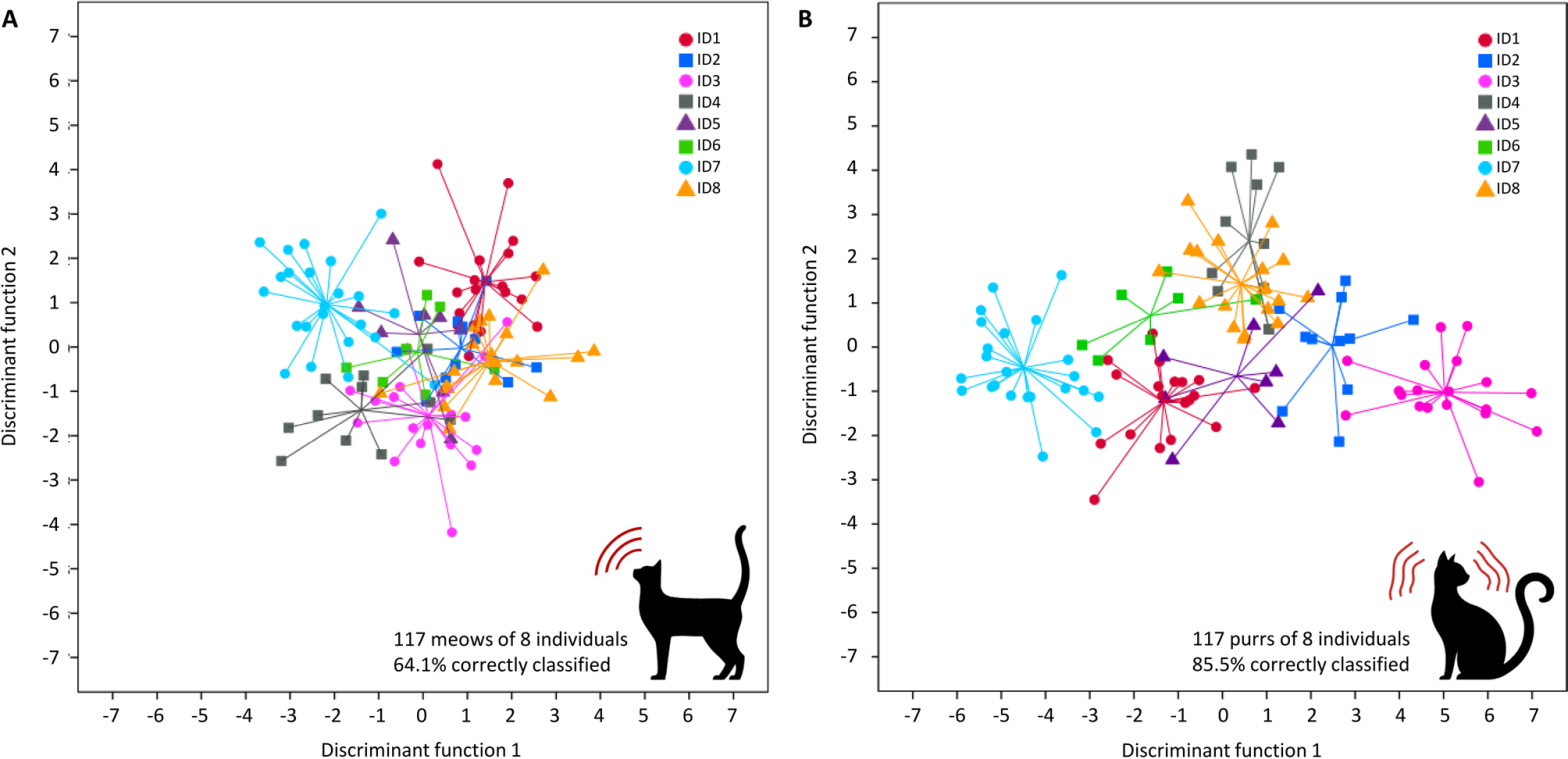
Individual signatures in meows (**A**) and purrs (**B**) of eight domestic cats. The two-dimensional signal space is defined by the first two discriminant functions, which were most important for individual discrimination. Each symbol marks the position of one meow or purr in signal space. Colour-symbol combinations represent different individuals. Lines converge on the respective centroids. Purrs encode a stronger individual signature than meows.

When calculating the DFA-based classification of equal numbers of meows and purrs from the same eight individuals (**Table ****S1**), our earlier findings were corroborated: 64.4% of meows and 85.5% of purrs were classified to the correct individual (Fig. 3; Tables 1 and 2), which was significantly better than a random classification (binomial tests: *p* < 0.001; 12.5% chance level for both vocalization types).

**Table 1:** Assessment of model fit for DFAs with equal numbers of meows and purrs from domestic cats.

	DF1	DF2	DF3	DF4	DF5	DF6	DF7
**Assessment of model fit - Meows**							
Eigenvalue	2.16	1.33	0.69	0.48	0.25	0.15	0.05
Explained variation [%]	42.4	26.0	13.5	9.4	4.8	2.9	0.9
Wilk’s Lambda	0.04	0.12	0.27	0.45	0.67	0.83	0.96
Chi-squared (for *p* < 0.05)	354.17	231.21	140.95	85.02	43.07	19.58	
**Assessment of model fit - Purrs**							
Eigenvalue	10.02	1.70	1.33	1.09	0.48	0.22	0.04
Explained variation [%]	67.3	11.4	8.9	7.3	3.2	1.5	0.3
Wilk’s Lambda	0.01	0.04	0.11	0.25	0.53	0.79	0.96
Chi-squared (for *p* < 0.05)	600.17	343.38	237.18	146.84	68.17	25.96	

**Table 2:** Classification matrix for DFAs with equal numbers of meows and purrs from domestic cats.

Cat ID	ID1	ID2	ID3	ID4	ID5	ID6	ID7	ID8	Number of recordings
	**Predicted ID [%] – Meows**								
ID1	**68.4**	5.3	0.0	0.0	10.5	0.0	0.0	15.8	19
ID2	0.0	**45.5**	0.0	9.1	9.1	0.0	0.0	36.4	11
ID3	5.3	0.0	**63.2**	21.1	5.3	5.3	0.0	0.0	19
ID4	0.0	0.0	0	**81.8**	0	0.0	9.1	9.1	11
ID5	0.0	12.5	12.5	12.5	**12.5**	0.0	37.5	12.5	8
ID6	0.0	14.3	14.3	0.0	0.0	**42.9**	14.3	14.3	7
ID7	0.0	0.0	0.0	4.3	4.3	4.3	**82.6**	4.3	23
ID8	10.5	10.5	10.5	0.0	0.0	5.3	0.0	**63.2**	19
	**Predicted ID [%] – Purrs**								
ID1	**89.5**	0	0	0	5.3	0	5.3	0	19
ID2	0	**81.8**	0	0	18.2	0	0	0	11
ID3	0	15.8	**84.2**	0	0	0	0	0	19
ID4	0	9.1	0	**63.6**	0	0	0	27.3	11
ID5	12.5	37.5	0	0	**50.0**	0	0	0	8
ID6	0	0	0	0	0	**100.0**	**0**	**0**	7
ID7	4.3	0	0	0	0	0	**95.7**	0	23
ID8	0	0	0	10.5	0	0	0	**89.5**	19

We used the H_S_ stereotypy index to assess how many different individuals can be encoded by each vocalisation type, as another way of comparing the relative strength of the individual signature in meows and purrs. The index provided additional support for a stronger individual signature in purrs than in meows: purrs may encode up to 22 individuals (2^4.47^ = 22.23), while meows can encode only up to six individuals (2^2.65^ = 6.26). Taken together, our results clearly show a stronger individual signature in purrs than in meows.

### Meow variability in domestic vs. wild cats

**Fig. 4:**
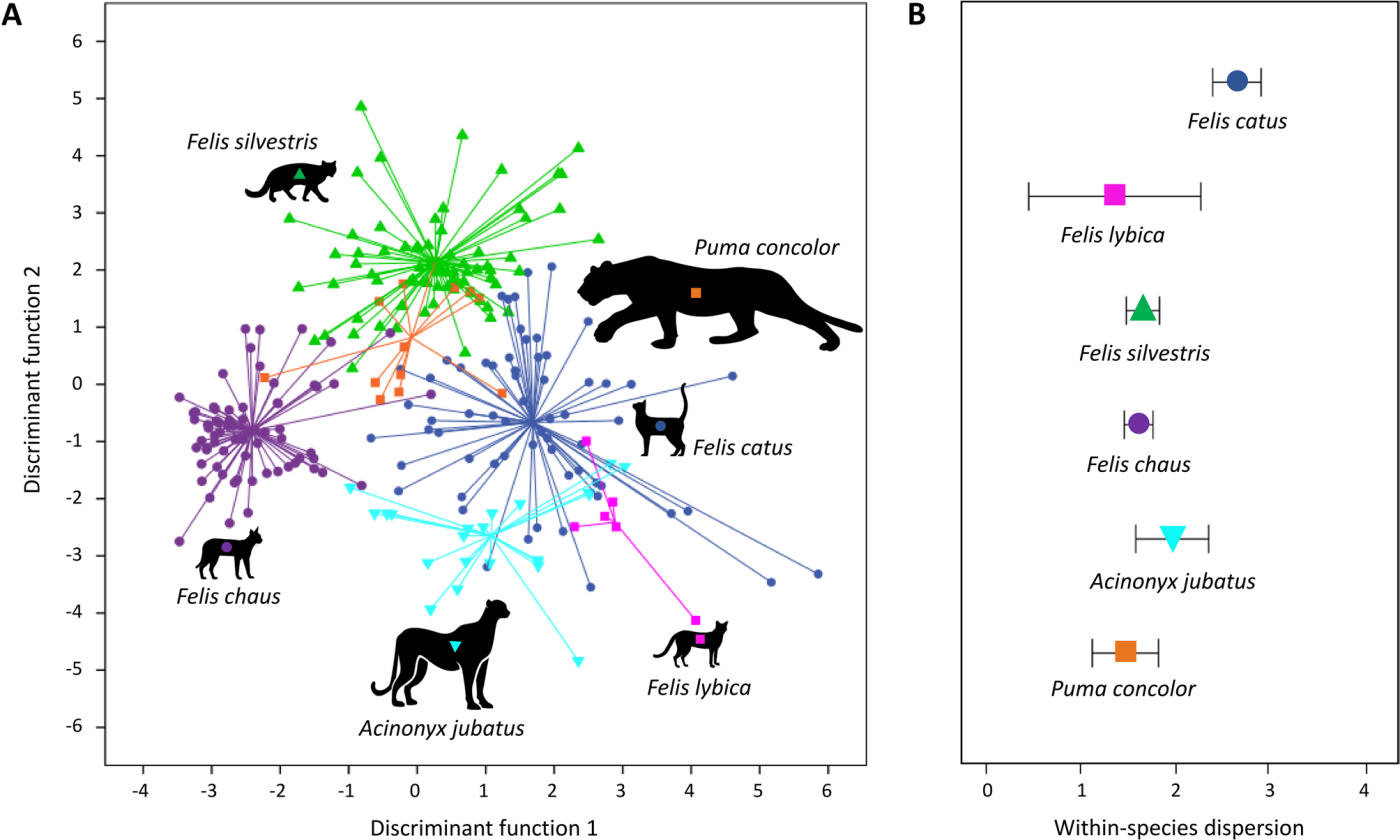
Species-specific differences in meows of eight domestic cats. (**A**) Each symbol in the two-dimensional signal space marks the position of one meow. Colour-symbol combinations correspond to different species. Lines converge on the respective centroids. (**B**) Meows of domestic cats have a higher within-species dispersion than meows of other cat species.

When comparing the meows of six cat species, there was a clear acoustic difference between species (Fig. 4A). The DFAs (see Table 3 for details on model fit and Table 4 for the classification matrix) classified 87.1% of meows to the correct species (binomial test: *p* < 0.001; 16.7% chance level). The within-species dispersion in the DFA signal space (i.e. the mean Euclidian distance between meows and their respective species centroids) indicates that domestic cats have much more variable meows than the other five cat species (Fig. 4B). The mean Euclidian distance in DFA signal space differed significantly between domestic cats and the other cat species (ANOVA; F_5_ = 15.294, *p* < 0.0001, partial eta squared = 0.24; all post-hoc tests comparing domestic cats to other cat species *p* < 0.01).

**Table 3:** Assessment of model fit for DFA with meows from six cat species.

Assessment of model fit	DF1	DF2	DF3	DF4	DF5
Eigenvalue	2.73	2.53	1.13	0.34	0.12
Explained variation [%]	39.9	36.8	16.5	5.0	1.8
Wilk’s l	0.02	0.09	0.31	0.66	0.89
Chi-squared (for *p* < 0.05)	894.65	579.87	278.78	97.70	27.25

**Table 4:** Classification matrix for DFA with meows from six cat species.

Species ID	Predicted Species ID [%]						Number of recordings
	ID1	ID2	ID3	ID4	ID5	ID6	
*F. catus*	**77.8**	0.0	0.0	1.4	0.0	7.7	63
*F. lybica*	3.2	**100.0**	0.0	0.0	4.5	0.0	6
*F. silvestris*	9.5	0.0	**93.3**	4.3	0.0	15.4	75
*F. chaus*	1.6	0.0	4.0	**94.2**	18.2	7.7	69
*A. jubatus*	6.3	0.0	0.0	0.0	**77.3**	0.0	22
*P. concolor*	1.6	0.0	2.7	0.0	0.0	**69.2**	13

## Discussion

We demonstrate that both meows and purrs in domestic cats provide reliable acoustic cues to individual identity, with purrs displaying significantly stronger signatures than meows. Moreover, the interspecific comparative findings indicate that domestication has substantially expanded meow variability, probably reflecting evolutionary trends toward enhanced human–animal communication. Together, these results highlight the sophisticated acoustic communication of domestic cats and their ongoing evolution within the human cultural environment.

Contrary to our initial hypothesis, it is the low-frequency, stereotyped purring that yields higher classification accuracy and carries markedly more individual information, as shown by discriminant analysis and the stereotypy index H_S_. Our finding is novel and somewhat counterintuitive, considering that meows, often seen as more socially flexible and context-dependent, are typically thought to be more informative during cat–human interactions^23,44^. While interindividual variation in some purr structural parameters had been previously noticed^28,29^, ours is the first study to demonstrate the strength of the individual signatures encoded in this vocalisation and its associated potential to convey a cat’s identity and characteristics. Our methodological approach, including the use of mel-frequency cepstral coefficients (MFCCs), proved effective in capturing identity-relevant features. MFCCs provide a holistic representation of vocal signals by summarising their overall spectral shape into a small set of coefficients, enabling direct comparison of acoustically distinct call types within a common analytical framework^45^.

Our study examined only the strength of individual signatures in purrs versus meows, and the stronger individual signature found in purrs does not necessarily imply a communicative function. Individual differences in animals’ vocal tract size, laryngeal anatomy, or neuromuscular control might naturally lead to stable acoustic differences^46–49^, even without selection for individuality. The domestic cat’s larynx capacity of producing purring sounds at characteristic frequencies without active neural input or muscle contraction indicates a largely anatomical origin, more closely tied to vocal tract morphology than meows^33^. If individual signatures do not reduce signal efficiency, there may be no evolutionary pressure to eliminate them; thus, individuality might persist as a neutral trait. This is especially likely since purrs are low-amplitude, short-range signals produced at distances where other sensory channels, including olfactory, tactile, and visual cues, likely dominate interactions^20,44,50^. However, even if other senses prevail at close range, multimodal cue redundancy might still be advantageous for recognising conspecifics, especially when environmental conditions make the other senses less effective.

Unlike purrs, meows may balance communication flexibility and recognisability. Meows’ modulation based on context, emotional valence, and learned associations likely reduces their acoustic consistency^35^. While purrs probably help with self-communication or bonding among conspecifics, the strong influence of human interaction and social reinforcement on meow structure^19,27,51^ may have increased their contextual variability, weakening consistent identity signals. This difference suggests that purring may reflect a “default” individualised acoustic profile, shaped mainly by the vocal tract in the absence of significant contextual or emotional influences. Conversely, the meow may have evolved through domestication to serve a communicative purpose where identity cues are less important than eliciting specific responses from humans. The acoustic variations in meows appear to be highly specific to cat-owner pairs, indicating they may result from ontogenetic ritualisation, an associative learning process in which cats and owners gradually shape the signal structure together during repeated interactions^20,52^. In such situations, owners already recognise the caller’s identity, which may reduce the evolutionary pressure for meows to encode identity.

Crucially, the cross-species comparison revealed that domestic cats exhibited significantly greater acoustic dispersion in meows than any of the five sampled wild felids. Specifically, domestic cats showed the greatest variation within species in meow structure, while the meows of species such as *F. silvestris*, *F. lybica*, and *F. chaus* were notably more stereotyped. This supports the hypothesis that domestication has increased vocal plasticity in the domestic cat’s repertoire, especially in meows, highlighting the role of human interaction in shaping these signals. Although rare in wild felines, meows in domestic cats have diversified considerably in structure and function as an adaptive response to anthropogenic social environments. Humans find domestic cat meows more pleasant than those of wild cats, and domestication may have exerted selective pressure on meows based on human preferences^51^. Cats were selected during domestication to modify features of their meows, such as fundamental frequency (F0) and vocalisation rate, demonstrating a high level of plasticity upon which human selection could act^51^. Especially in context-rich call types like the meow, increased vocal variability may have evolved after domestication as part of a broader shift in communication towards human responsiveness, which also involved changes in vocal tract morphology. Emerging evidence suggests that domestication influences vocal output in animals not only behaviourally but also morphologically, as recent studies link changes in vocalisations to neural crest cell development, affecting the larynx^53^.

In conclusion, we show that domestication has reshaped the acoustic communication of cats in complex ways, amplifying the variability of human-directed signals while preserving strong individual signatures in low-amplitude, close-range vocalisations. This dual pattern suggests that human-driven selection and the demands of cat–human interaction have diversified the vocal repertoire and influenced how individual information is expressed across call types. By linking individual signatures, vocal plasticity, and domestication within a single framework, our study provides a foundation for a better understanding of how domestic animals adapt their communication systems to the unique selective pressures of human-dominated environments.

## Supplementary Information

Below is the link to the electronic supplementary material.

Supplementary Material 1

Supplementary Material 2

## Data Availability

All data in this manuscript is available in the electronic supplementary material.
