# Supplementary material for: Meows encode less individual information than purrs and show greater variability in domestic than in wild cats
Source: Sci Rep. 2025 Dec 9;15:43490. doi: 10.1038/s41598-025-31536-7 (PMC12695941; doi:10.1038/s41598-025-31536-7)
Supplement: Supplementary file 1 — Supplementary Material 1 [file 41598_2025_31536_MOESM1_ESM.docx]

**Electronical Supplementary Material**

**Meows encode less individual information than purrs and show greater variability in domestic than in wild cats**

Danilo Russo, Anja Schild, Mirjam Knörnschild

Content: Data S1, Table S1-S4

**Data S1:**

Sheet 1: MFCC, duration, and spectral centroid for meows of domestic cats.

Sheet 2: MFCC, duration, and spectral centroid for purrs of domestic cats.

Sheet 3: MFCC, duration, and spectral centroid for meows of six cat species.

**Table S1**: Individual cats in study.

| **cat name** | **cat sex** | **cat breed** | **# meows** | **# purrs** | **in balanced data set** |
| --- | --- | --- | --- | --- | --- |
| Attikus | male | European Shorthair | 0 | 20 | no |
| Blacky | female | European Shorthair | 12 | 0 | no |
| Chili | male | European Shorthair | 0 | 10 | no |
| Coco | female | European Shorthair | 0 | 33 | no |
| Danillo | male | Birman | 19 | 39 | yes |
| Diane | female | Birman | 17 | 11 | yes |
| Elvis | male | Maine Coon Mix | 31 | 0 | no |
| Ernie | male | Neva Masquerade | 0 | 10 | no |
| Floh | female | European Shorthair | 0 | 48 | no |
| Frederik | male | European Shorthair Mix | 8 | 0 | no |
| Gretchen | female | European Shorthair | 0 | 19 | no |
| Hugo | male | European Shorthair | 19 | 30 | yes |
| Karim | male | European Shorthair | 11 | 23 | yes |
| Kelly | female | British Shorthair | 0 | 56 | no |
| Lilly | female | British Shorthair | 0 | 37 | no |
| Linus | male | British Shorthair | 8 | 43 | yes |
| Louis | male | European Shorthair | 15 | 0 | no |
| Luna | female | European Shorthair | 0 | 11 | no |
| Marek | male | European Shorthair | 0 | 28 | no |
| Mia | female | British Shorthair | 0 | 33 | no |
| Mina | female | Siam Mix | 0 | 38 | no |
| Mio | male | European Shorthair | 53 | 0 | no |
| Paul | male | European Shorthair | 7 | 7 | yes |
| Wookie | male | European Shorthair | 0 | 19 | no |
| Yoda | male | British Shorthair | 29 | 23 | yes |
| Zeus | male | Norwegian Forest Cat | 21 | 19 | yes |
| Zorro | male | Maine Coon Mix | 26 | 0 | no |

**Table S2**: Assessment of model fit for two DFAs with 276 meows and 557 purrs.

| **Assessment of model fit  - Meows** | **DF1** | **DF2** | **DF3** | **DF4** | **DF5** | **DF6** | **DF7** | **DF8** | **DF9** | **DF10** |
| --- | --- | --- | --- | --- | --- | --- | --- | --- | --- | --- |
| Eigenvalue | 1.74 | 1.09 | 1.01 | 0.74 | 0.41 | 0.35 | 0.20 | 0.12 | 0.07 | 0.02 |
| explained variation [%] | 30.2 | 19.0 | 17.4 | 13.0 | 7.2 | 6.0 | 3.4 | 2.1 | 1.1 | 0.4 |
| Wilk's l | 0.02 | 0.05 | 0.10 | 0.21 | 0.36 | 0.51 | 0.69 | 0.82 | 0.92 | 0.98 |
| Chi-squared (for p < 0.05) | 1056.6 | 792.0 | 598.2 | 415.0 | 268.7 | 177.6 | 99.3 | 51.8 | 21.9 |  |
| **Assessment of model fit  - Purrs** | **DF1** | **DF2** | **DF3** | **DF4** | **DF5** | **DF6** | **DF7** | **DF8** | **DF9** | **DF10** |
| Eigenvalue | 3.46 | 2.46 | 1.70 | 1.00 | 0.94 | 0.72 | 0.24 | 0.20 | 0.14 | 0.07 |
| explained variation [%] | 31.4 | 22.2 | 15.4 | 10.2 | 8.5 | 6.5 | 2.2 | 1.8 | 1.3 | 0.5 |
| Wilk's l | 0.01 | 0.01 | 0.03 | 0.08 | 0.17 | 0.32 | 0.56 | 0.69 | 0.83 | 0.95 |
| Chi-squared (for p < 0.05) | 3392.1 | 2583.4 | 1912.9 | 1375.4 | 968.0 | 609.5 | 317.4 | 200.0 | 102.0 | 29.4 |

**Table S3**: Classification matrix for a DFA with 276 meows from 14 individuals.

| **Cat  ID** | **Predicted ID [%] - Meows** | | | | | | | | | | | | | | **Number of** |  |
| --- | --- | --- | --- | --- | --- | --- | --- | --- | --- | --- | --- | --- | --- | --- | --- | --- |
|  | **ID1** | **ID2** | **ID3** | **ID4** | **ID5** | **ID6** | **ID7** | **ID8** | **ID9** | **ID10** | **ID11** | **ID12** | **ID13** | **ID14** | **recordings** | |
| ID1 | **66.7** | 8.3 | 0.0 | 8.3 | 0.0 | 0.0 | 0.0 | 0.0 | 0.0 | 8.3 | 0.0 | 0.0 | 8.3 | 0.0 | 12 | |
| ID2 | 0.0 | **68.4** | 15.8 | 0.0 | 0.0 | 5.3 | 0.0 | 0.0 | 5.3 | 0.0 | 0.0 | 5.3 | 0.0 | 0.0 | 19 | |
| ID3 | 0.0 | 23.5 | **41.2** | 0.0 | 0.0 | 11.8 | 0.0 | 5.9 | 0.0 | 5.9 | 5.9 | 0.0 | 5.9 | 0.0 | 17 | |
| ID4 | 0.0 | 0.0 | 0.0 | **83.9** | 0.0 | 0.0 | 0.0 | 0.0 | 0.0 | 12.9 | 0.0 | 3.2 | 0.0 | 0.0 | 31 | |
| ID5 | 0.0 | 0.0 | 0.0 | 25.0 | **37.5** | 0.0 | 25.0 | 0.0 | 0.0 | 0.0 | 0.0 | 0.0 | 12.5 | 0.0 | 8 | |
| ID6 | 5.3 | 5.3 | 5.3 | 0.0 | 0.0 | **52.6** | 15.8 | 0.0 | 0.0 | 15.8 | 0.0 | 0.0 | 0.0 | 0.0 | 19 | |
| ID7 | 0.0 | 0.0 | 0.0 | 9.1 | 0.0 | 0.0 | **54.5** | 0.0 | 9.1 | 9.1 | 0.0 | 0.0 | 0.0 | 18.2 | 11 | |
| ID8 | 0.0 | 0.0 | 37.5 | 0.0 | 0.0 | 12.5 | 0.0 | **12.5** | 0.0 | 0.0 | 0.0 | 25.0 | 12.5 | 0.0 | 8 | |
| ID9 | 0.0 | 0.0 | 0.0 | 6.7 | 0.0 | 0.0 | 0.0 | 0.0 | **53.3** | 40.0 | 0.0 | 0.0 | 0.0 | 0.0 | 15 | |
| ID10 | 0.0 | 1.9 | 0.0 | 9.4 | 0.0 | 0.0 | 5.7 | 0.0 | 0.0 | **71.7** | 1.9 | 3.8 | 1.9 | 3.8 | 53 | |
| ID11 | 0.0 | 0.0 | 28.6 | 14.3 | 14.3 | 0.0 | 0.0 | 0.0 | 0.0 | 28.6 | **0.0** | 14.3 | 0.0 | 0.0 | 7 | |
| ID12 | 0.0 | 0.0 | 0.0 | 0.0 | 3.4 | 0.0 | 0.0 | 0.0 | 0.0 | 13.8 | 0.0 | **72.4** | 3.4 | 6.9 | 29 | |
| ID13 | 4.8 | 0.0 | 9.5 | 4.8 | 0.0 | 0.0 | 0.0 | 0.0 | 0.0 | 4.8 | 0.0 | 0.0 | **76.2** | 0.0 | 21 | |
| ID14 | 0.0 | 0.0 | 0.0 | 3.8 | 0.0 | 0.0 | 0.0 | 0.0 | 0.0 | 3.8 | 0.0 | 3.8 | 0.0 | **88.5** | 26 | |

**Table S4**: Classification matrix for a DFA with 557 purrs from 21 individuals.

| **Cat ID** | **Predicted ID [%] - Purrs** | | | | | | | | | | | | | | | | | | | | | **Number of recordings** |
| --- | --- | --- | --- | --- | --- | --- | --- | --- | --- | --- | --- | --- | --- | --- | --- | --- | --- | --- | --- | --- | --- | --- |
|  | **ID1** | **ID2** | **ID3** | **ID4** | **ID5** | **ID6** | **ID7** | **ID8** | **ID9** | **ID10** | **ID11** | **ID12** | **ID13** | **ID14** | **ID15** | **ID16** | **ID17** | **ID18** | **ID19** | **ID20** | **ID21** |  |
| ID1 | **80.0** | 0.0 | 0.0 | 0.0 | 5.0 | 0.0 | 0.0 | 5.0 | 0.0 | 0.0 | 0.0 | 0.0 | 0.0 | 0.0 | 0.0 | 0.0 | 10.0 | 0.0 | 0.0 | 0.0 | 0.0 | 20 |
| ID2 | 0.0 | **80.0** | 0.0 | 0.0 | 0.0 | 0.0 | 0.0 | 0.0 | 0.0 | 0.0 | 0.0 | 0.0 | 0.0 | 20.0 | 0.0 | 0.0 | 0.0 | 0.0 | 0.0 | 0.0 | 0.0 | 10 |
| ID3 | 0.0 | 0.0 | **72.7** | 0.0 | 0.0 | 0.0 | 21.2 | 3.0 | 0.0 | 0.0 | 0.0 | 0.0 | 0.0 | 0.0 | 0.0 | 0.0 | 0.0 | 0.0 | 3.0 | 0.0 | 0.0 | 33 |
| ID4 | 0.0 | 0.0 | 0.0 | **76.9** | 2.6 | 0.0 | 7.7 | 0.0 | 0.0 | 0.0 | 2.6 | 0.0 | 0.0 | 0.0 | 10.3 | 0.0 | 0.0 | 0.0 | 0.0 | 0.0 | 0.0 | 39 |
| ID5 | 0.0 | 0.0 | 0.0 | 0.0 | **63.6** | 0.0 | 0.0 | 0.0 | 0.0 | 0.0 | 9.1 | 0.0 | 18.2 | 0.0 | 0.0 | 0.0 | 9.1 | 0.0 | 0.0 | 0.0 | 0.0 | 11 |
| ID6 | 0.0 | 0.0 | 0.0 | 0.0 | 0.0 | **70.0** | 10.0 | 0.0 | 0.0 | 0.0 | 0.0 | 0.0 | 0.0 | 0.0 | 0.0 | 0.0 | 0.0 | 0.0 | 20.0 | 0.0 | 0.0 | 10 |
| ID7 | 0.0 | 0.0 | 0.0 | 2.1 | 2.1 | 0.0 | **77.1** | 0.0 | 0.0 | 0.0 | 0.0 | 0.0 | 2.1 | 6.3 | 0.0 | 0.0 | 6.3 | 0.0 | 4.2 | 0.0 | 0.0 | 48 |
| ID8 | 0.0 | 0.0 | 0.0 | 0.0 | 0.0 | 5.3 | 5.3 | **63.2** | 0.0 | 5.3 | 0.0 | 0.0 | 0.0 | 0.0 | 0.0 | 0.0 | 15.8 | 0.0 | 0.0 | 0.0 | 5.3 | 19 |
| ID9 | 0.0 | 0.0 | 0.0 | 0.0 | 6.7 | 0.0 | 0.0 | 0.0 | **93.3** | 0.0 | 0.0 | 0.0 | 0.0 | 0.0 | 0.0 | 0.0 | 0.0 | 0.0 | 0.0 | 0.0 | 0.0 | 30 |
| ID10 | 0.0 | 0.0 | 0.0 | 0.0 | 0.0 | 0.0 | 26.1 | 0.0 | 0.0 | **56.5** | 0.0 | 8.7 | 0.0 | 0.0 | 0.0 | 0.0 | 8.7 | 0.0 | 0.0 | 0.0 | 0.0 | 23 |
| ID11 | 0.0 | 0.0 | 0.0 | 0.0 | 1.8 | 0.0 | 3.6 | 0.0 | 0.0 | 0.0 | **82.1** | 0.0 | 0.0 | 0.0 | 0.0 | 8.9 | 1.8 | 1.8 | 0.0 | 0.0 | 0.0 | 56 |
| ID12 | 0.0 | 0.0 | 0.0 | 0.0 | 0.0 | 0.0 | 2.7 | 0.0 | 0.0 | 2.7 | 0.0 | **86.5** | 0.0 | 0.0 | 0.0 | 2.7 | 5.4 | 0.0 | 0.0 | 0.0 | 0.0 | 37 |
| ID13 | 0.0 | 0.0 | 0.0 | 0.0 | 7.0 | 0.0 | 0.0 | 0.0 | 0.0 | 0.0 | 0.0 | 0.0 | **74.4** | 0.0 | 2.3 | 0.0 | 14.0 | 0.0 | 0.0 | 2.3 | 0.0 | 43 |
| ID14 | 0.0 | 45.5 | 0.0 | 18.2 | 0.0 | 0.0 | 9.1 | 0.0 | 0.0 | 0.0 | 0.0 | 0.0 | 0.0 | **9.1** | 0.0 | 18.2 | 0.0 | 0.0 | 0.0 | 0.0 | 0.0 | 11 |
| ID15 | 0.0 | 3.6 | 0.0 | 3.6 | 0.0 | 0.0 | 14.3 | 0.0 | 0.0 | 0.0 | 0.0 | 0.0 | 0.0 | 0.0 | **75.0** | 0.0 | 0.0 | 0.0 | 3.6 | 0.0 | 0.0 | 28 |
| ID16 | 0.0 | 0.0 | 0.0 | 3.0 | 0.0 | 0.0 | 6.1 | 0.0 | 0.0 | 0.0 | 6.1 | 0.0 | 0.0 | 3.0 | 0.0 | **81.8** | 0.0 | 0.0 | 0.0 | 0.0 | 0.0 | 33 |
| ID17 | 0.0 | 0.0 | 0.0 | 0.0 | 2.6 | 0.0 | 0.0 | 0.0 | 0.0 | 0.0 | 0.0 | 0.0 | 5.3 | 0.0 | 0.0 | 0.0 | **92.1** | 0.0 | 0.0 | 0.0 | 0.0 | 38 |
| ID18 | 0.0 | 0.0 | 0.0 | 0.0 | 0.0 | 0.0 | 0.0 | 0.0 | 0.0 | 0.0 | 0.0 | 0.0 | 0.0 | 0.0 | 0.0 | 0.0 | 0.0 | **100.0** | 0.0 | 0.0 | 0.0 | 7 |
| ID19 | 0.0 | 0.0 | 0.0 | 0.0 | 0.0 | 0.0 | 26.3 | 0.0 | 0.0 | 0.0 | 0.0 | 5.3 | 0.0 | 0.0 | 0.0 | 0.0 | 0.0 | 0.0 | **68.4** | 0.0 | 0.0 | 19 |
| ID20 | 0.0 | 0.0 | 0.0 | 4.3 | 0.0 | 0.0 | 0.0 | 4.3 | 0.0 | 0.0 | 0.0 | 0.0 | 0.0 | 0.0 | 8.7 | 0.0 | 0.0 | 0.0 | 0.0 | **82.6** | 0.0 | 23 |
| ID21 | 0.0 | 0.0 | 0.0 | 0.0 | 0.0 | 0.0 | 5.3 | 5.3 | 0.0 | 10.5 | 0.0 | 0.0 | 5.3 | 0.0 | 0.0 | 0.0 | 26.3 | 0.0 | 0.0 | 0.0 | **47.4** | 19 |
